# Magnesium levels and outcome after allogeneic hematopoietic stem cell transplantation in acute myeloid leukemia

**DOI:** 10.1007/s00277-020-04382-y

**Published:** 2020-12-19

**Authors:** Linus Angenendt, Isabel Hilgefort, Jan-Henrik Mikesch, Bernhard Schlüter, Wolfgang E. Berdel, Georg Lenz, Matthias Stelljes, Christoph Schliemann

**Affiliations:** 1 Department of Medicine A, University Hospital Münster, Albert-Schweitzer-Campus 1, 48149 Münster, Germany; 2 Centre for Laboratory Medicine, University Hospital Münster, Münster, Germany

**Keywords:** Acute myeloid leukemia, Allogeneic hematopoietic stem cell transplantation, GVHD, GVL, Magnesium

## Abstract

Low intake of magnesium has been associated with the occurrence of lymphomas and decreased magnesium levels suppress the cytotoxic function of T cells and natural killer cells in patients with “X-linked immunodeficiency with magnesium defect, Epstein-Barr virus infection, and neoplasia” (XMEN) syndrome. These cell types are also important mediators of immune-mediated effects after allogeneic hematopoietic stem cell transplantation. Here, we show that high posttransplant magnesium levels independently associate with a lower incidence of relapse, a higher risk of acute graft-versus-host disease, and a higher non-relapse mortality in 368 patients with acute myeloid leukemia from our center. Magnesium serum levels might impact on donor-cell-mediated immune responses in acute myeloid leukemia.

**Supplementary Information:**

The online version contains supplementary material available at 10.1007/s00277-020-04382-y.

## Introduction

Allogeneic hematopoietic stem cell transplantation (HSCT) is an effective treatment option for patients with hematological malignancies, such as acute myeloid leukemia (AML). Here, it offers reduced rates of relapse when performed in first complete remission and is the best option for recurrent disease [1, 2]. However, it comes at an increased risk for life-threatening donor-cell-mediated acute graft-versus-host disease (GVHD) and infections due to conditioning and posttransplantation immunosuppression.

Patients with “X-linked immunodeficiency with magnesium defect, Epstein-Barr virus (EBV) infection, and neoplasia” (XMEN), resulting from a loss-of-function mutation in the *magnesium transporter 1* (*MAGT1*), have low intracellular free magnesium levels and an increased risk for malignancies and chronic EBV infections [3]. Decreased intracellular free magnesium has recently been shown to suppress the cytotoxic functions of natural killer cells and cytotoxic T lymphocytes in these patients [4], two cell types that are important mediators of the graft-versus-leukemia effect and GVHD. Magnesium supplementation in patients with XMEN syndrome led to restoration of the cytotoxic function of these cells and clearance of EBV infection [4]. In addition, an association of magnesium depletion with the development of non-Hodgkin lymphomas has been reported [5]. Interestingly, deficiency of magnesium is a common finding following allogeneic HSCT, resulting from metabolic derangements and calcineurin inhibitor–associated renal magnesium wasting [6]. In this retrospective analysis, we hypothesized that posttransplant magnesium levels may associate with outcome after HSCT in patients with AML.

## Methods

### Patients, samples, and treatment

Patients who received their first HSCT for the treatment of AML at the University Hospital Münster, Germany, between 2008 and 2016, achieved leukocyte engraftment, and had serum magnesium levels measured during the posttransplant period were included in this study. The study was approved by the local institutional review board (2016-576-f-S). HSCT, GVHD prophylaxis, and supportive care were performed according to local standard operating procedures and as previously described [7].

Serum magnesium was quantified using commercially available photometric assays on Roche/Hitachi cobas c (Roche Diagnostics GmbH, Mannheim, Germany) oder ADVIA Chemistry Systems (Siemens Healthcare Diagnostics Inc., Tarrytown, NY, USA). Viremia was determined by polymerase chain reaction (PCR) using the artus CMV QS-RGQ Kit and the artus EBV QS-RGQ Kit (both Quiagen, Hilden, Germany). All measurements were performed with accredited laboratory methods at the Center of Laboratory Medicine of the University Hospital Münster.

### Definition of endpoints and statistical analyses

Mean posttransplant magnesium levels were calculated from the day of HSCT until the day of relapse or death without relapse in every patient individually who had an event within first 100 days after HSCT. In patients who did not experience an event within 100 days, mean magnesium levels were calculated individually for each patient from the day of HSCT until the median time to relapse or death within the first 100 days from this cohort, which was 39 days. Thus, magnesium values measured after a given event were not included for calculation of average magnesium levels. Patients were divided into those with low and high posttransplant magnesium values at the median. Mean posttransplant ciclosporin serum concentrations were calculated the same way as magnesium values for each patient.

Baseline patient characteristics were compared using Mann-Whitney test for continuous and *χ*^2^ or Fisher’s exact test for categorical variables. Time-to-event variables were measured from day of HSCT until death for overall survival (OS), hematological relapse for cumulative incidence of relapse (CIR), and death without prior relapse for cumulative incidence of non-relapse mortality (NRM). The cumulative incidence of acute GVHD was calculated from day of HSCT until acute GVHD with prior relapse and death as competing events. Survival probabilities were determined with the Kaplan-Meier method and in case of competing events (CIR, NRM, and acute GVHD) with the Aalen-Johansen estimator. All survival probabilities are given at 3 years. Follow-up time was calculated by the reverse Kaplan-Meier method. Multivariable Cox and Fine-Gray regression models were constructed to assess the significance of prognostic factors with respect to OS, CIR, NRM, and acute GVHD as appropriate. The prognostic impact of posttransplant magnesium values was adjusted for age, disease status before HSCT, HLA compatibility, cytomegalovirus (CMV) risk constellation, and ciclosporin serum concentration. The consistency of association with outcomes across subgroups was examined with test for interaction. The proportional hazards assumption was verified for each variable individually by inspection of scaled Schoenfeld residuals. Missing data were not imputed. Two-sided *p* values < 0.05 were considered significant. All analyses were performed using the statistical environment R (www.r-project.org), version 4.0.0.

## Results

### Patient characteristics

A total of 368 patients were identified. Serum magnesium values were available for most of the days from HSCT until the respective endpoint, with a median availability of 75.0% of those days (IQR, 67.5–85.0%). Median of mean posttransplant serum magnesium levels was 0.76 mmol/l (range, 0.59–1.00 mmol/l). Baseline patient and transplant characteristics according to magnesium levels are shown in Table 1. High magnesium levels were significantly associated with older age (*p* = 0.0007) and secondary AML (*p* = 0.013).

**Table 1:** Disease and transplant characteristics of 368 AML patients

Variables	Magnesium level		*p* value
	Low	High	
*N*	184	184	
Age (years)			**.0007**^§^
Median (range)	53 (18–74)	58 (18–74)	
Sex, no. (%)			.67^¶^
Male	103 (56.0)	108 (58.7)	
Female	81 (44.0)	76 (41.3)	
AML type, no. (%)			**.013**^‡^
De novo	151 (82.1)	129 (70.1)	
s-AML	27 (14.7)	50 (27.2)	
t-AML	6 (3.3)	5 (2.7)	
FAB, no. (%)			.64^‡^
M0	14 (8.9)	18 (11.7)	
M1	25 (15.8)	24 (15.6)	
M2	38 (24.1)	32 (20.8)	
M4	37 (23.4)	41 (26.6)	
M5	35 (22.2)	36 (23.4)	
M6	8 (5.1)	3 (1.9)	
M7	1 (0.6)	0 (0.0)	
Unknown	26 (14.1)	30 (16.3)	
*FLT3*-ITD, no. (%)			.37^¶^
Present	40 (21.9)	32 (17.6)	
Absent	143 (78.1)	150 (82.4)	
Unknown	1 (0.5)	2 (1.1)	
*NPM1*, no. (%)			.31^¶^
Mutated	45 (24.6)	36 (19.7)	
Wild type	138 (75.4)	147 (80.3)	
Unknown	1 (0.5)	1 (0.5)	
Cytogenetic and molecular risk*, no. (%)			.14^¶^
Favorable	35 (19.2)	38 (20.7)	
Intermediate	110 (60.4)	94 (51.1)	
Adverse	37 (20.3)	52 (28.3)	
Unknown	2 (1.1)	0 (0.0)	
HCT-CI score**, no. (%)			.49^¶^
0–2	155 (84.2)	149 (81.0)	
≥ 3	29 (15.8)	35 (19.0)	
Disease status before HSCT***, no. (%)			.53^¶^
No active disease	96 (52.2)	89 (48.4)	
Active disease	88 (47.8)	95 (51.6)	
Conditioning, no. (%)			.55^‡^
RIC	79 (42.9)	69 (37.5)	
SEQ	96 (52.2)	106 (57.6)	
MAC	9 (4.9)	9 (4.9)	
Stem cell source, no. (%)			.28^‡^
PBSC	182 (98.9)	178 (96.7)	
BM	2 (1.1)	6 (3.3)	
Donor, no. (%)			.37^‡^
MRD	57 (31.0)	49 (26.6)	
MMRD	0 (0.0)	1 (0.5)	
MUD	96 (52.2)	92 (50.0)	
MMUD	28 (15.2)	40 (21.7)	
Haploidentical	3 (1.6)	2 (1.1)	
Recipient-donor sex match, no. (%)			.22^¶^
Female donor—male recipient	28 (15.2)	38 (20.7)	
Other	156 (84.8)	146 (79.3)	
CMV risk, no. (%)			.74^¶^
R+	118 (64.1)	122 (66.3)	
R−	66 (35.9)	62 (33.7)	

*AML*, acute myeloid leukemia; *s-AML*, secondary AML; *t-AML*, therapy-related AML; *FAB*, French-American-British classification; *FLT3-ITD*, internal tandem duplication of the *FLT3* gene; *NPM1*, nucleophosmin-1; *HSCT*, hematopoietic stem cell transplantation; *NMA*, non-myeloablative conditioning; *RIC*, reduced intensity conditioning; *SEQ*, sequential conditioning; *MAC*, myeloablative conditioning; *PBSC*, peripheral blood stem cells; *BM*, bone marrow; *MRD*, matched related donor; *MMRD*, mismatched related donor; *MUD*, matched unrelated donor; *MMUD*, mismatched unrelated donor; *R+*, recipient positive; *R−*, recipient negative. Significant *p* values are marked in bold

*According to European LeukemiaNet 2010 guidelines (information on CEBPA mutational status not available) [9]

**According to Sorror et al. [10]

***No active disease includes patients with complete remission (CR), CR with incomplete hematological recovery (CRi), or morphologic leukemia-free state (MLFS) and active disease patients with ≥ 5% bone marrow blasts

^§^Mann-Whitney test; ^¶^chi-square test; ^‡^Fisher’s exact test

### Magnesium levels and outcome

**Fig. 1:**
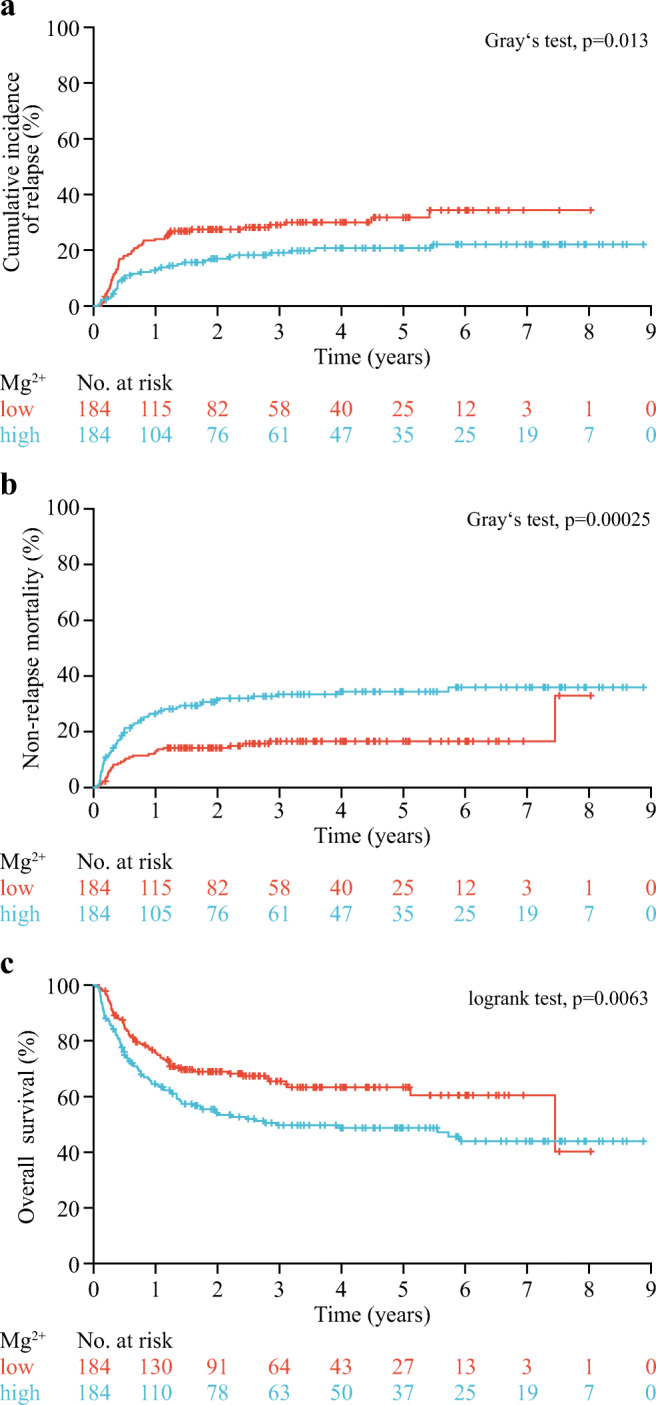
Magnesium levels and outcome in AML patients after allogeneic HSCT. Cumulative incidence of relapse (**a**), non-relapse mortality (**b**), and overall survival (**c**) according to posttransplant serum magnesium levels

**Fig. 2:**
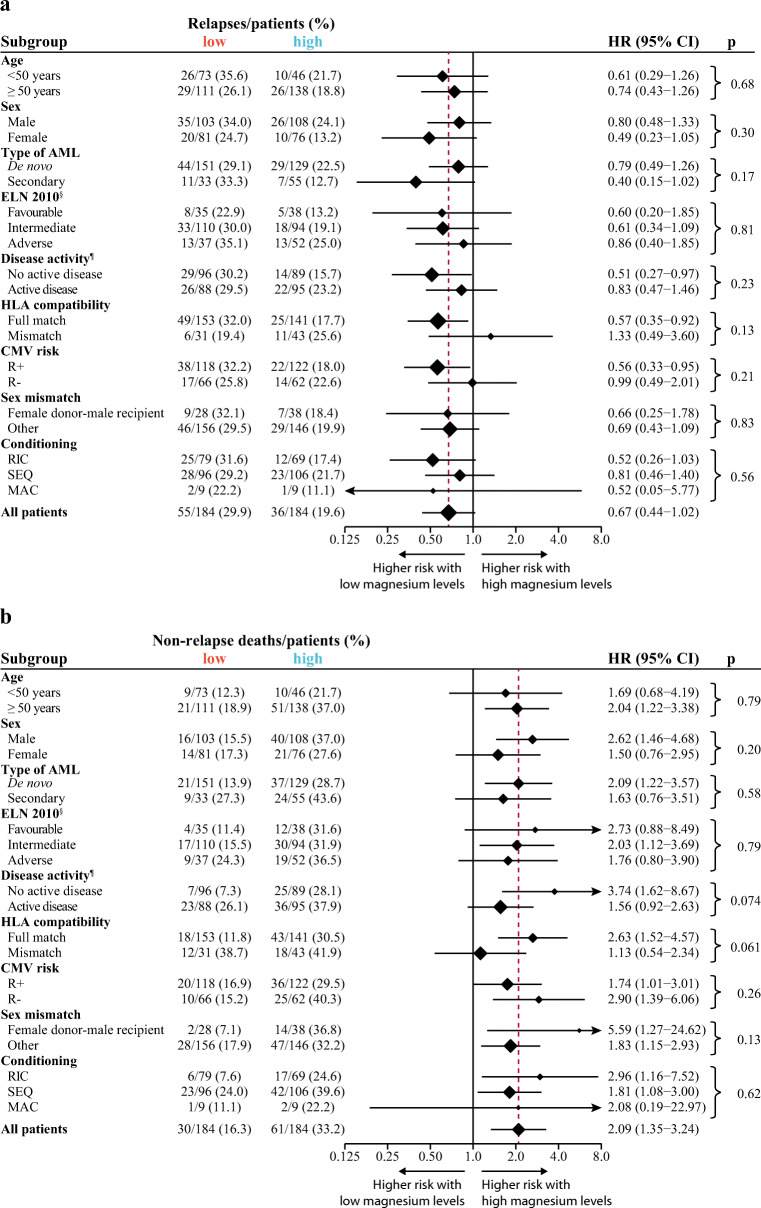
Cumulative incidence of relapse (**a**) and non-relapse mortality (**b**) according to posttransplant serum magnesium values by selected baseline categories. Diamonds represent the pooled unadjusted hazard ratios (HRs). Horizontal lines represent the 95% confidence intervals (CIs). The dotted vertical line represents the HR from the complete cohort. The *p* values are for interaction of unadjusted HRs by subgroups and represent heterogeneity. ^§^According to European LeukemiaNet (ELN) 2010 guidelines (information on CEBPA mutational status not available). ^¶^No active disease includes patients with complete remission (CR), CR with incomplete hematological recovery or morphologic leukemia-free state (MLFS), and active disease patients with ≥ 5% bone marrow blasts. AML, acute myeloid leukemia; HLA, human leukocyte antigen; CMV, cytomegalovirus; R+, recipient positive; R-, recipient negative; RIC, reduced intensity conditioning; SEQ, sequential conditioning; MAC, myeloablative conditioning

Median follow-up was 3.65 years (95% confidence interval [CI], 3.29–4.03 years). High posttransplant serum magnesium levels were significantly associated with a lower CIR (19.0% vs 29.1%, for high vs low; *p* = 0.013; Fig. 1a) and a higher NRM (33.5% vs 16.6%; *p* = 0.00025; Fig. 1b). Overall, high magnesium levels were associated with inferior OS (49.7% vs 65.4%; *p* = 0.0063; Fig. 1c). Magnesium levels remained associated with CIR (hazard ratio [HR], 0.62; 95% CI, 0.41–0.93; *p* = 0.023) and NRM (HR, 1.95; 95% CI, 1.27–3.00; *p* = 0.0025) after adjustment for age, disease status before HSCT, HLA compatibility, CMV risk, and ciclosporin serum levels (Table 2). Overall, patients with high posttransplant magnesium levels had a significantly higher risk of death (HR, 1.44; 95% CI, 1.04–2.01; *p* = 0.029) after adjustment for the same variables. We found no significant heterogeneity of the association of posttransplant serum magnesium values with CIR and NRM by age, sex, type of AML, genetic risk, disease status before allogeneic HSCT, HLA compatibility, CMV risk group, sex mismatch, and conditioning regimens (Fig. 2).

**Table 2:** Multivariable regression analyses

Variables	HR	95% CI	*p* value
Cumulative incidence of relapse			
Age: per 10-year increase	0.94	0.81–1.10	.45
Disease status before HSCT^§^: active disease vs no active disease	1.20	0.80–1.81	.37
HLA compatibility: mismatch vs full match	0.89	0.53–1.48	.66
CMV risk: R+ vs R−	1.05	0.69–1.60	.81
Ciclosporin: high vs low	1.00	0.66–1.53	.99
Magnesium: high vs low	0.62	0.41–0.93	.023
Non-relapse mortality			
Age: per 10-year increase	1.15	0.94–1.40	.17
Disease status before HSCT^§^: active disease vs no active disease	2.05	1.34–3.14	.00090
HLA compatibility: mismatch vs full match	1.93	1.24–3.01	.0035
CMV risk: R+ vs R−	0.82	0.54–1.25	.36
Ciclosporin: high vs low	0.67	0.44–1.03	.067
Magnesium: high vs low	1.95	1.27–3.00	.0025
Overall survival			
Age: per 10-year increase	1.03	0.89–1.18	.72
Disease status before HSCT^§^: active disease vs no active disease	1.98	1.43–2.75	< .0001
HLA compatibility: mismatch vs full match	1.63	1.13–2.34	.0086
CMV risk: R+ vs R−	1.05	0.75–1.47	.79
Ciclosporin: high vs low	0.80	0.58–1.11	.18
Magnesium: high vs low	1.44	1.04–2.01	.029

Hazard ratios (HRs) greater or less than 1.0 indicate an increased or decreased risk, respectively, of an event per increase of the continuous variables and for the first category listed for the categorical variables. Collinearity among predictors was low with a variance inflation factor of 1.07 (range, 1.00–1.20). *HSCT*, hematopoietic stem cell transplantation; *RIC*, reduced intensity conditioning; *SEQ*, sequential conditioning; *MAC*, myeloablative conditioning; *HLA*, human leukocyte antigen; *CMV*, cytomegalovirus

^§^Active disease includes patients with ≥ 5% bone marrow blasts and no active disease patients with CR, CR with incomplete hematological recovery (CRi), or morphologic leukemia-free state (MLFS)

**Fig. 3:**
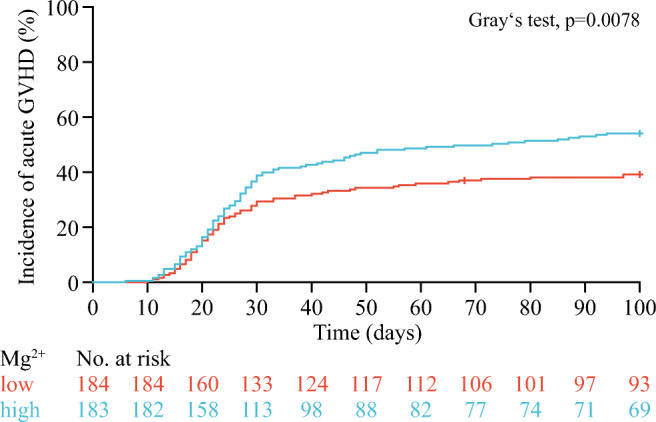
Cumulative incidence of acute GVHD according to posttransplant serum magnesium values

Rates of acute GVHD were significantly higher in patients with high magnesium levels (54.9% vs 38.3%; *p* = 0.0020). To confirm the association of magnesium levels with incidence of acute GVHD, we recalculated mean posttransplant magnesium values and mean ciclosporin concentrations from the day of HSCT until the day of the occurrence of acute GVHD, relapse, or death, whichever occurred first, in patients who experienced such an event within 100 days after HSCT. For patients not experiencing an event within 100 days after HSCT, mean magnesium values and ciclosporin concentrations were calculated until the median time to the occurrence of such an event within 100 days after HSCT in this cohort, which was 27 days. Using these values, and dichotomization at the median, we confirmed that the cumulative incidence of acute GVHD was higher in patients with high than with low posttransplant magnesium levels (54.1% vs 39.2%; *p* = 0.0078; Fig. 3). Patients with high magnesium levels had a significantly higher risk of acute GVHD than patients with low levels (HR, 1.43; 95% CI, 1.04–1.97; *p* = 0.027; Table 3) after multivariable adjustment for age, disease status before HSCT, HLA compatibility, CMV risk, and ciclosporin serum concentration.

**Table 3:** Multivariable regression analyses

Variables	HR	95% CI	*p* value
Cumulative incidence of acute GVHD			
Age: per 10-year increase	0.91	0.80–1.04	.15
Disease status before HSCT^§^: active disease vs no active disease	1.17	0.86–1.59	.31
HLA compatibility: mismatch vs full match	1.90	1.33–2.73	.0005
CMV risk: R+ vs R−	1.23	0.89–1.69	.21
Ciclosporin: high vs low	1.15	0.84–1.56	.39
Magnesium: high vs low	1.43	1.04–1.97	.027

Hazard ratios (HRs) greater or less than 1.0 indicate an increased or decreased risk, respectively, of an event per increase of the continuous variables and for the first category listed for the categorical variables. Collinearity among predictors was low with a variance inflation factor of 1.06 (range, 1.03–1.12). *GVHD*, graft-versus-host disease; *HSCT*, hematopoietic stem cell transplantation; *HLA*, human leukocyte antigen; *CMV*, cytomegalovirus; *R+*, recipient positive; *R−*, recipient negative

^§^Active disease includes patients with ≥ 5% bone marrow blasts and no active disease patients with complete remission (CR), CR with incomplete hematological recovery (CRi), or morphologic leukemia-free state (MLFS)

Given that calcineurin inhibitors may cause hypomagnesemia by suppressing reabsorption of magnesium from renal tubules and thus low magnesium levels may reflect high ciclosporin exposure, we analyzed associations between ciclosporin (used for GVHD prophylaxis in all patients) and magnesium levels. Magnesium levels did not correlate with ciclosporin levels when calculated as described above or until onset of acute GVHD (*r* = − 0.058, *p* = 0.27 and *r* = − 0.0036, *p* = 0.95, respectively; supplemental Figure S1 and S2). Most importantly, mean ciclosporin serum levels (calculated the same way as magnesium levels) were not prognostic for CIR, NRM, acute GVHD, or OS (all *p* > 0.05).

As a sensitivity analysis, mean magnesium and ciclosporin values were added as a continuous variable to the multivariable models. Here, associations remained significant for OS, NRM, and risk of acute GVHD (supplemental Table S1 and S2). In addition, we recalculated individual posttransplant serum magnesium levels as above, using the median instead of the mean. Again, high magnesium levels were significantly associated with inferior OS (50.2% vs 64.7%; *p* = 0.0060), higher cumulative incidences of NRM (32.2% vs 17.8%; *p* = 0.0015), and acute GVHD (54.6% vs 37.4%; *p* = 0.0022), and in trend lower CIR (20.6% vs 27.7%; *p* = 0.087) (supplemental Figure S3).

Finally, we found no associations of magnesium levels with the cumulative incidence of CMV or EBV viremia during the first 100 days after transplantation (*p* = 0.83 and 0.82, respectively).

## Discussion

Deficiency of magnesium is a common finding following HSCT, resulting from metabolic derangements and calcineurin inhibitor–associated renal magnesium wasting, among other reasons, and magnesium is often supplemented in these patients. In patients with XMEN syndrome, magnesium supplementation has been described to upregulate NKG2D expression on and restore the cytotoxic function of NK and T cells and lead to EBV clearance [4]. The receptor NKG2D has also been shown to regulate acute GVHD and graft-versus-leukemia effects after allogeneic HSCT [8]. The association of high magnesium levels with a lower CIR and a higher NRM as well as a higher risk of acute GVHD in our study might suggest an impact of posttransplant magnesium levels on donor-cell-mediated alloimmune responses. Overall, however, the net effect of high posttransplant magnesium levels was connected with inferior OS.

Our study has several limitations. First, in theory, posttransplant serum magnesium levels could be inversely associated with ciclosporin levels, so the impact on CIR, NRM, and risk of acute GVHD would simply be explained by the degree of ciclosporin-induced immunosuppression. However, we found no correlation of magnesium and ciclosporin values in our cohort. Furthermore, there was no association of ciclosporin levels with all endpoints analyzed, and the association of magnesium levels with outcome remained after adjustment for ciclosporin levels in multivariable models. Second, other drugs such as antibiotics or the presence of diarrhea and vomiting could result in lower magnesium values, while renal dysfunction might associate with higher magnesium values. However, information on these variables during the posttransplant period was not available. Likewise, while higher magnesium serum levels might reflect the degree of magnesium supplementation, data on magnesium supplementation in individual patients were not available. However, according to our hypothesis, magnesium levels as a whole, whether supplemented or not, should have an influence on immune effector cells. The predominance of really measured over averaged magnesium data points, covering 75% of all days preceding an event of interest, further supports the validity of our analyses.

In summary, we show that posttransplant serum magnesium levels are associated with outcome in AML after allogeneic HSCT. Our results might provide a rationale for the design of a prospective low interventional clinical trial that tests the degree of magnesium supplementation after allogeneic HSCT, especially in the context of acute GVHD. Additional laboratory investigations are needed to clarify the effects of magnesium on donor-cell-mediated immune responses against leukemic cells.

## Supplementary information

ESM 1 (PDF 322 kb)
