# Supplementary material for: Magnesium levels and outcome after allogeneic hematopoietic stem cell transplantation in acute myeloid leukemia
Source: Ann Hematol. 2020 Dec 19;100(7):1871–8. doi: 10.1007/s00277-020-04382-y (PMC8195955; doi:10.1007/s00277-020-04382-y)

Magnesium Levels and Outcome After Allogeneic Hematopoietic Stem Cell Transplantation in Acute Myeloid Leukemia

Linus Angenendt[1], Isabel Hilgefort[1], Jan-Henrik Mikesch[1], Bernhard Schlüter[2], Wolfgang E. Berdel[1], Georg Lenz[1], Matthias Stelljes[1], Christoph Schliemann[1]

[1]*Department of Medicine A, University Hospital Münster, Münster, Germany.*
[2]*Centre for Laboratory Medicine, University Hospital Münster, Münster, Germany.*

Supplemental Tables

Supplemental Table S1. Multivariable regression analyses using magnesium as a continuous variable.

Variables	HR	95% CI	p-value
**Cumulative incidence of relapse**			
Age: per 10-year increase	0.95	0.81-1.11	.52
Disease status before HSCT[§]: active disease vs no active disease	1.19	0.80-1.78	.39
HLA compatibility: mismatch vs full match	0.87	0.53-1.44	.60
CMV risk: R+ vs R-	1.03	0.68-1.56	.90
Ciclosporin: per 10-unit increase	0.97	0.89-1.06	.48
Magnesium: per 0.1-unit increase	0.74	0.48-1.14	.17
**Non-relapse mortality**			
Age: per 10-year increase	1.13	0.94-1.37	.20
Disease status before HSCT[§]: active disease vs no active disease	2.08	1.36-3.16	.00066
HLA compatibility: mismatch vs full match	1.97	1.25-3.12	.0037
CMV risk: R+ vs R-	0.82	0.54-1.25	.36
Ciclosporin: per 10-unit increase	0.96	0.88-1.05	.36
Magnesium: per 0.1-unit increase	1.88	1.27-2.78	.0017
**Overall survival**			
Age: per 10-year increase	1.01	0.89-1.16	.83
Disease status before HSCT[§]: active disease vs no active disease	1.97	1.42-2.74	<.0001
HLA compatibility: mismatch vs full match	1.64	1.14-2.36	.0081
CMV risk: R+ vs R-	1.03	0.73-1.44	.88
Ciclosporin: per 10-unit increase	0.96	0.90-1.02	.22
Magnesium: per 0.1-unit increase	1.55	1.15-2.08	.0038

Hazard ratios (HR) greater or less than 1.0 indicate an increased or decreased risk, respectively, of an event per increase of the continuous variables and for the first category listed for the categorical variables. Collinearity among predictors was low with a variance inflation factor of 1.07 (range, 1.00-1.20). Abbreviations: HSCT, hematopoietic stem cell transplantation; RIC, reduced intensity conditioning; SEQ, sequential conditioning; MAC, myeloablative conditioning; HLA, human leukocyte antigen; CMV, cytomegalovirus.

[§]Active disease includes patients with ≥ 5% bone marrow blasts and no active disease patients with CR, CR with incomplete hematological recovery (CRi) or morphologic leukemia free state (MLFS).

Supplemental Table S2. Multivariable regression analysis for acute GVHD with magnesium as a continuous variable.

Variables	HR	95% CI	p-value
**Cumulative incidence of acute GVHD**			
Age: per 10-year increase	0.90	0.79-1.03	.14
Disease status before HSCT§: active disease vs no active disease	1.18	0.87-1.60	.28
HLA compatibility: mismatch vs full match	1.89	1.33-2.69	.00037
CMV risk: R+ vs R-	1.27	0.92-1.74	.14
Ciclosporin: per 10-unit increase	1.01	0.95-1.06	.84
Magnesium per 0.1-unit increase	1.51	1.16-1.96	.0021

Hazard ratios (HR) greater or less than 1.0 indicate an increased or decreased risk, respectively, of an event per increase of the continuous variables and for the first category listed for the categorical variables. Collinearity among predictors was low with a variance inflation factor of 1.06 (range, 1.03-1.12). Abbreviations: GVHD, graft-versus-host disease; HSCT, hematopoietic stem cell transplantation; HLA, human leukocyte antigen; CMV, cytomegalovirus; R+, recipient positive; R-, recipient negative.

§Active disease includes patients with ≥ 5% bone marrow blasts and no active disease patients with complete remission (CR), CR with incomplete hematological recovery (CRi) or morphologic leukemia free state (MLFS).

Supplemental Figures

Supplemental Figure S1. Correlation of mean ciclosporin serum concentrations and mean posttransplant magnesium serum values, both measured until relapse, death or median time until relapse or death.

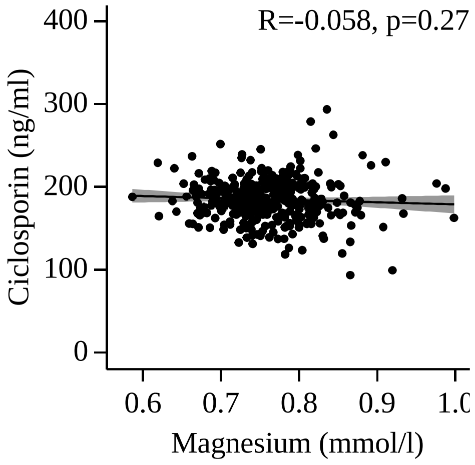

Supplemental Figure S2. Correlation of mean ciclosporin serum concentrations and mean posttransplant magnesium serum values, both measured until acute GVHD, relapse, death or median time until acute GVHD, relapse or death.

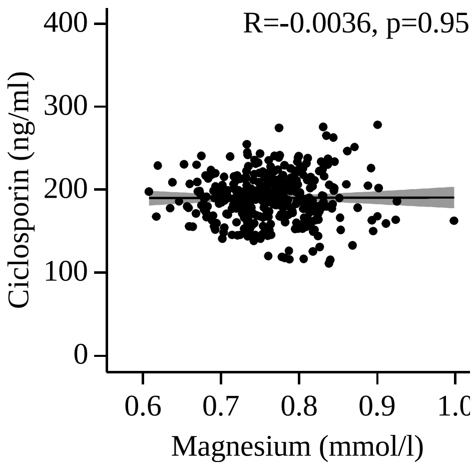

Supplemental Figure S3. Median magnesium levels and outcome in AML patients after allogeneic HSCT. Cumulative incidence of relapse (CIR), non-relapse mortality (NRM), overall survival and cumulative incidence of acute GVHD according to median posttransplant serum magnesium levels.

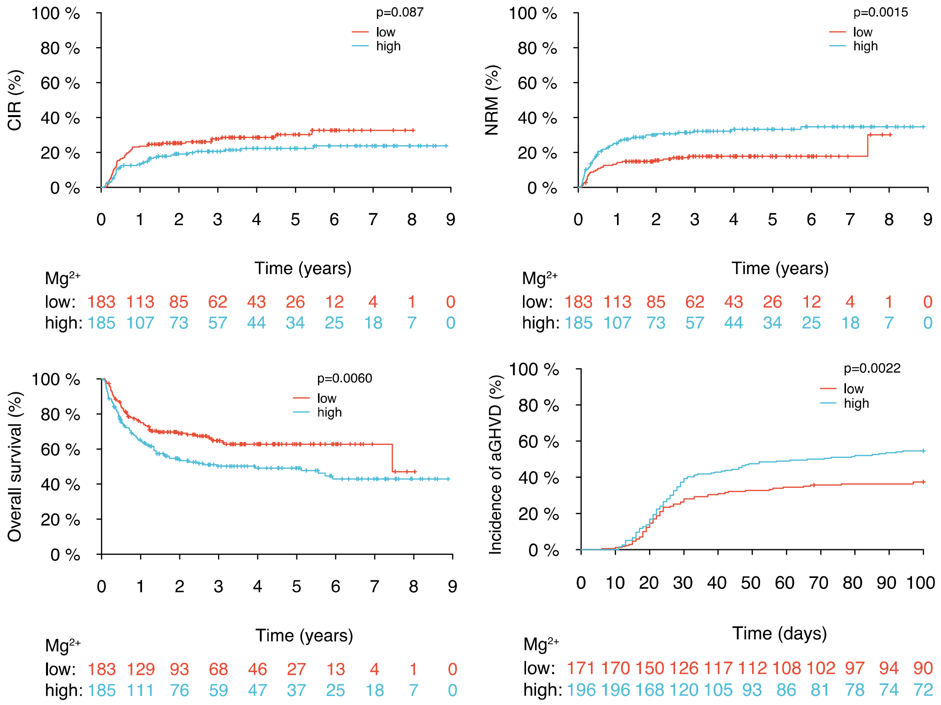

Supplement: Supplementary file 1 — (PDF 322 kb) [file 277_2020_4382_MOESM1_ESM.pdf]
